# Hydroxychloroquine-Induced Myopathy Responding to Intravenous Immunoglobulin (IVIG)

**DOI:** 10.7759/cureus.41016

**Published:** 2023-06-27

**Authors:** Hani Almoallim, Alaa Samkari, Ahmad Fallata, Heba Adam, Malak Kary, Mohammed Bahabri, Mohamed Cheikh

**Affiliations:** 1 Department of Medicine, Faculty of Medicine, Umm Al-Qura University, Makkah, SAU; 2 Department of Medicine, Faculty of Medicine, International Medical Center, Jeddah, SAU; 3 Department of Medicine, Faculty of Medicine, King Saud Bin Abdulaziz University for Health Sciences, Jeddah, SAU; 4 Department of Pathology and Laboratory Medicine, Faculty of Medicine, Ministry of National Guard-Health Affairs, Jeddah, SAU; 5 Department of Medicine, Faculty of Medicine, University of Tabuk, Tabuk, SAU; 6 Department of Medicine, Faculty of Medicine, Dr. Soliman Fakeeh Hospital, Jeddah, SAU; 7 Department of Medicine, Faculty of Medicine, King Abdulaziz University, Jeddah, SAU; 8 Department of Medicine, Faculty of Medicine, Fakeeh College for Medical Sciences, Jeddah, SAU

**Keywords:** myopathy, hydroxychloroquine, systemic lupus erythematosus, ivig, hydroxychloroquine-induced myopathy

## Abstract

Hydroxychloroquine (HCQ), a drug used to treat many diseases such as rheumatoid arthritis (RA) and systemic lupus erythematosus (SLE), has limited reports documenting drug-induced myopathies as a side effect. This entity is underdiagnosed with unclear treatment interventions apart from discontinuing the offending drug. We report a case of a biopsy-proven hydroxychloroquine-induced myopathy in a 35-year-old female patient with SLE. The offending drug was stopped, but the patient did not improve. However, she showed marked improvement after the use of intravenous immunoglobulin (IVIG).

## Introduction

Many commonly prescribed medications are myopathic, such as glucocorticoids, antimalarial drugs, statins, and fibrates. Therefore, it is crucial to know when to suspect this side effect. Drug-induced myopathy is defined as an acquired myopathy that can be asymptomatic or manifested by myopathic symptoms and signs, with or without elevated creatine kinase (CK) level, in patients without previous primary muscular diseases after their exposure to a certain drug [1,2]. Hydroxychloroquine (HCQ) was initially developed to treat malaria; meantime, it has a wide range of clinical applications. It is one of the most used disease-modifying anti-rheumatic drugs (DMARD) in clinical practice. It is used in all stages of systemic lupus erythematosus (SLE) and as a first-line DMARD in mild rheumatoid arthritis (RA) [2,3]. It has other uses; however, all are still off-label [3], and it was a candidate therapy during the COVID-19 pandemic [4].

Retinopathy is one of the adverse effects of HCQ and also chloroquine (CQ) that requires immediate discontinuation of the drug [3]. As such, it is heavily monitored among clinicians, compared to other side effects, such as cardiac and neuromuscular toxicities. The HCQ mechanism of action may play a major role in developing myopathy. It works by increasing the pH in intracellular vacuoles, altering protein degradation [4]. Furthermore, they inhibit lysosomal function, which alters the degradation of accumulated phospholipids and glycogen, resulting in curvilinear bodies, which are nuclear or cytoplasmic aggregates of proteins in a lamellated and twisted structure surrounded by a membrane. These bodies are implicated in the development of myopathy [5].

HCQ-induced myopathy has historically been a rare diagnosis, but recent publications show that it is an underdiagnosed and undertreated side effect [3]. Apart from discontinuing the drug, there is no major intervention reported in the literature to reverse the myopathy. We report a case of HCQ-induced myopathy with normal creatine kinase (CK) level in a long-standing lupus case. The diagnosis was confirmed by muscle biopsy. Her condition improved significantly after the use of intravenous immunoglobulins (IVIG).

## Case presentation

A 35-year-old Saudi female patient known to have SLE for 13 years was on HCQ 200 mg once daily since the diagnosis was made and prednisolone (PD) 15 mg once daily. Her disease was always limited to musculoskeletal and hematological manifestations. She had no major organ involvement such as the kidney nor neuropsychiatric manifestations of lupus. She presented to the rheumatology clinic in August 2020, complaining of knee and hand joint pain bilaterally. Her examination was remarkable for knee and wrist joint tenderness, without swelling, redness, or hotness. Laboratory investigations were remarkable for leukopenia of 2.0×10^3^/µL (normal count: 4.5-11×10^3^/µL), C-reactive protein (CRP) of 11 mg/L (normal count: <5 mg/L), erythrocyte sedimentation rate (ESR) of 23 mm/hour (normal range: 0-20 mm/hour), anti-double-stranded deoxyribonucleic acid (dsDNA) of 548 IU/ml (normal count: <27 IU/ml), low C3 of 0.65 g/L (normal count: 0.9-1.8 g/L), normal C4, and normal urinalysis and kidney function tests. She was treated as an active SLE manifested as arthritis, and adjustments for her medication were done as follows: 50 mg azathioprine (AZA) was added, HCQ dose was increased to 200 mg twice per day (BID), and her current PD dose of 15 mg once daily was tapered with 5 mg decrease for each week.

Two weeks later, at her next follow-up visit, she had worsened joint pain in the hand and tenderness all over her hand joints on examination. Therefore, naproxen 250 mg was added, AZA dose was increased to 100 mg per day, and there was no change in the tapered PD dose. Subsequent follow-up visits demonstrated improvement in her arthritis symptoms. However, her leukopenia was getting worse, reaching 1.63×10^3^/µL. AZA dose was changed to 100 mg per day alternating with 50 mg initially, and then, it was discontinued. Her symptoms after that recurred, and her laboratory results showed a persistent elevation of anti-dsDNA of >666 IU/ml. For that reason, she was started on intravenous belimumab (BLM) at an initial dose of 10 mg/kg every two weeks for three doses and then a maintenance dose of 10 mg/kg every four weeks, and she was kept on 5 mg/day PD and HCQ 200 mg twice per day. During the following visits and only after two doses of BLM, she had progressive proximal painless muscle weakness of the upper and lower limbs. It was continued with no diurnal variation. It was symmetric, and it was not associated with myalgia or muscle cramps.

Her symptoms were progressive to the extent that it interfered with her daily activities. She had no fever or any symptoms that indicate a focus of infection, no shortness of breathing, no dysphagia, and no skin rashes. She had no history of Raynaud's phenomenon. Past history was unremarkable for a preexisting disease that causes myopathy such as myasthenia gravis and metabolic and inherited disorders. She had no recent infection, no seizure, or no recent heavy exercise. There was no history of alcohol or other substance abuse. A general examination of the patient was negative for any rashes on the upper and lower limbs including extensor surfaces. She had no rashes on the eyelids and no rashes on the upper chest or anterior neck. Neurological examination revealed a power of 3/5 of the proximal muscles with intact distal power in the four limbs, sensation, and deep tendon reflexes. Cardiovascular, respiratory, and musculoskeletal examinations were unremarkable. Her laboratory examination did not differ from before and showed persistent leukopenia with persistent elevation of anti-dsDNA. There was no electrolyte imbalance. Her previously done CRP and ESR were repeated, and both were stationary. Her muscle enzyme CK total and lactate dehydrogenase (LDH) were both normal. Her thyroid and parathyroid function test was normal. She had normal liver function tests and normal viral serologies including HIV. Peripheral blood smear showed leukopenia and lymphopenia with no presence of schistocytes, and she had negative Coombs test. Her cardiac enzymes, ECG, and echocardiogram were insignificant. Anti-ribonucleoprotein (RNP) antibody was negative.

**Figure 1:**
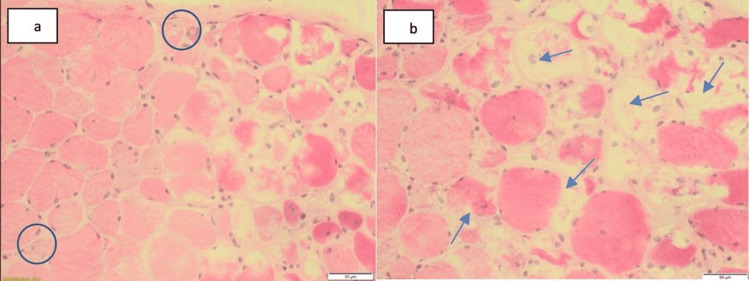
H&E-stained frozen specimen a) Skeletal muscle fibers with obvious variation in fiber size and scattered small rounded to angulated fibers (circles). b) Several large and small vacuoles (arrows)

**Figure 2:**
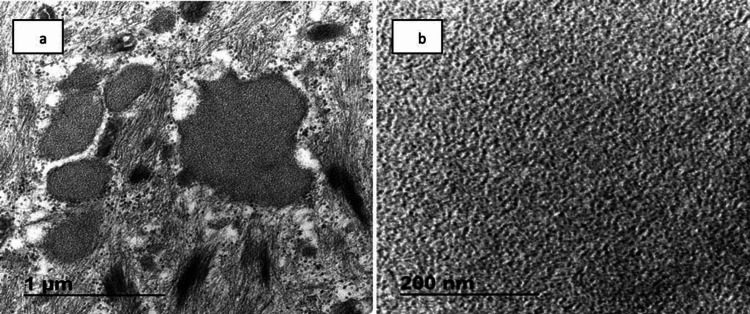
Electron microscopy a) Skeletal muscle with myofibrillar disarray and Z-line streaming. b) Curvilinear-like bodies identified

Neurological workup revealed normal MRI of the brain with normal cerebrospinal fluid chemistry and no albumin-cytologic dissociation. MRI of the thigh showed muscle inflammation in T2 signal. Electromyography showed rapid recruitment and myotonic discharges, which were suggestive of myositis with normal nerve conduction study (NCS). Muscle biopsy from her left thigh was arranged and showed necrosis of the muscle fibers with no inflammatory cells, several large and small vacuoles, and normal blood vessels. Electron microscopy showed myofibrillar disarray, Z-line streaming, and curvilinear-like bodies (Figures 1-2). The findings were not consistent with any type of inflammatory myopathy, and it was highly suggestive of drug-induced myopathy. Therefore, HCQ was discontinued without a significant recovery of her power, and BLM was not resumed. A decision was made to start her on intravenous immunoglobulins (IVIG), and the patient showed marked improvement of her symptoms from the first dose. Her improvement continued to reach almost full power on her proximal muscles (5/5). She received a total of six doses of IVIG (2 g/kg) on a monthly basis.

## Discussion

We reported a case of a female patient with long-standing SLE who was treated with HCQ, given its effectiveness and safety profile in females of childbearing age [1]. However, she presented with SLE flare, and while she was being followed up for that, she developed significant myopathy. Initially, we thought that it was attributable to her primary illness; therefore, HCQ was continued, and other medications such as AZA and BLM were added aiming to control her relapse. Despite the use of all these medications, she continued to have symptoms. It was a diagnostic predicament. Following the standard approaches in evaluating muscle weakness, we ultimately reached the diagnosis of HCQ-induced myopathy based on histopathological findings. Treatment, on the other hand, was another challenge as the patient did not improve after HCQ discontinuation, and her power was still severely impaired. In the literature, we found a reported case of SLE that presented with myalgia and fatigue; she was treated as SLE flared and ended up with quadriparesis; at that time, a diagnosis of HCQ-induced myopathy was established [2]. This encouraged us to proceed with IVIG as a last resort, which resulted in a remarkable outcome of complete reversibility of the HCQ myotoxic effect.

HCQ has many side effects ranging from mild to serious. Mild side effects including gastrointestinal and various cutaneous manifestations are common and require no treatment interruption [3]. Serious side effects are retinopathy, myopathy including cardiomyopathy, nephropathy, and neurological and psychiatric side effects. Some are described in most of the publications in the literature as rare, and others have few reported cases [3,4]. Myopathy is one of the side effects that is considered rare, but many studies recently are claiming that it is rather an underdiagnosed entity [6,7]. There is a definite difficulty facing physicians diagnosing HCQ-induced myopathy. The case presented here is a typical example of such a diagnostic dilemma one can pass through. Because of that, articles reporting its prevalence are few, and data are fluctuating. A review article reported the incidence of HCQ-induced myopathy to be 12.6%; another prospective study of 119 patients reported 6.7% as the prevalence of HCQ-induced myopathy [4,8]. Until now, there is no established prevalence of HCQ-induced myopathy [6]. On the contrary, the ocular toxicity of HCQ has an established prevalence as it has clear guidelines for screening and monitoring [9]. This is probably what makes it easy for clinicians to assess, and clearly, this is not the situation with HCQ-induced myopathy.

It is believed that the myopathy caused by HCQ can be understood by the molecular structure of the drug and its mechanism of action. Antimalarial drugs are highly lipophilic, have extensive tissue uptake, and have long terminal half-life [10]. Furthermore, they inhibit lysosomal function, which alters the degradation of accumulated phospholipids and glycogen, resulting in curvilinear bodies. Other signs present in histopathology, such autophagic vacuoles and pathological changes to mitochondria, support this hypothesis [5]. These findings were most helpful and consistent in the histopathological assessment of our case (Figures 1-2).

The risk factors for HCQ-induced myopathy are Caucasian race and renal failure [3]. Age-related decrease in muscle mass and strength and the coadministrations of other drugs could be additional risk factors. A systemic review and meta-analysis found that the majority of patients with antimalarial-induced myopathy are females who are above 50 years old [7]. Another retrospective cohort study reported the presence of curvilinear bodies in 67% of patient with HCQ-induced myopathy, who were taking proton pump inhibitors (PPI) [11]. Our reported patient here is a female with an intact kidney function, but she was taking PPI as prophylactic therapy. Certain factors might play a role in the development of HCQ-induced myopathy; nonetheless, definitive risk factors are not yet entirely clear.

Another interesting finding in this case report is the normal levels of CK and lactate dehydrogenase in the presence of significant myopathy. A systemic review and meta-analysis reported that the majority of patients that had a biopsy-proven antimalarial-induced myopathy have elevated CK total level [7]. In the contrary, the patient might be asymptomatic with elevated muscle enzymes [7]. Elevated CK total was higher in CQ-induced myopathy than HCQ-induced myopathy in one study [7]. This means that the normal levels of muscle enzymes cannot exclude the presence of myopathy, and rather, we should consider them a complementary tool in any patient suspected to have antimalarial-induced myopathy.

It is known in clinical practice that once a drug-induced side effect is established, it should be immediately discontinued as we had done in our case. However, the patient's condition unfortunately did not improve for few weeks. One of the side effects of PD is myopathy; hence, increasing its dose in our situation will lead to more confusion; we did not change our plan of keeping her on a tapered steroid dose. After a long discussion with neurology service, we decided to proceed with the use of IVIG. It was administered at a rate of 2 g/kg for a total of six doses on a monthly basis. Surprisingly, the patient demonstrated an impressive clinical outcome of complete regaining of her muscle power. Finally, she finished her IVIG course with no side effects.

IVIG is rarely used for drug-induced myopathy. Three patients with statin-induced autoimmune myopathy responded well to IVIG [12]. The mechanism of action that is responsible for this improvement is not clear. An anti-inflammatory and/or immunomodulatory effect of the drug might play a role [13]. Our finding suggests that IVIG may attenuate antimalarial-induced myopathy, but it should be used cautiously especially in patients who are at risk for thromboembolic events. Obviously, more evidence is needed before considering IVIG as a treatment for HCQ-induced myopathy. This evidence might be hard to be obtained without strict vigilance for this underdiagnosed entity.

## Conclusions

HCQ-induced myopathy reflects an underdiagnosed rather than rare side effect of the drug with serious consequences. In clinical practice, we still lack data for monitoring this side effect and, when encountered, how it should be treated. However, muscle biopsy is an essential step to diagnose it. Aside from stopping the offending drug, IVIG might be a treatment option. It is not easy to design studies to address these issues.
